# Revision of the family Carabodidae (Acari, Oribatida) VII. Redefinition of the genus *Malgasodes*; redescription of *M. curvisetus* Mahunka, 2000; and complementary description of *M. hungarorum* Mahunka, 2010. Phylogenetic relationships between Malgasodes, *Bovicarabodes*, *Afticarabodes*, *Congocepheus* and *Cavaecarabodes* are discussed

**DOI:** 10.3897/zookeys.435.8071

**Published:** 2014-08-15

**Authors:** Nestor Fernandez, Pieter Theron, Christine Rollard, Elio Rodrigo Castillo

**Affiliations:** 1 National Council of Scientific and Technological Research, Argentina (CONICET). Subtropical Biological Institut (IBS). Evolutive Genetic Laboratory FCEQyN, Misiones National University. Felix de Azara 1552, 6°, (3300) Posadas Misiones Argentina; 2 Research Unit for Environmental Sciences and Management, North-West University, Potchefstroom Campus, 2520, South Africa; 3 Muséum National d’Histoire Naturelle, Département Systématique et Evolution, Unité OSEB, Section Arthropodes, 57 rue Cuvier. 75231, Paris cedex 05. France

**Keywords:** Acari, Oribatida, Carabodidae, *Malgasodes*, taxonomy, phylogenetic relationships

## Abstract

The genus *Malgasodes* is redefined; the type species *M. curvisetus* Mahunka, 2000, is redescribed by means of studies using optic and Scanning Electron Microsopy (SEM), and a complementary description of *M. hungarorum* Mahunka, 2000 is included. Comparison of genera *Malgasodes* Mahunka, 2000, *Bovicarabodes* Fernandez, Theron, Rollard, 2013a, *Cavaecarabodes* Fernandez, Theron, Rollard, Rodriguez Castillo, 2014, *Afticarabodes* Fernandez, Theron, Rollard, 2013b, and *Congocepheus* Balogh, 1958 is made. Problems concerning chaetotaxy, regressive evolution and neotrichy are explained and phylogenetic relationships between *Malgasodes*, *Bovicarabodes*, *Afticarabodes*, *Congocepheus* and *Cavaecarabodes* are discussed.

## Introduction

For several years we have been working on a revision of the family Carabodidae. Many years ago, upon commencing our work on this family, we studied large collections of material, principally from Madagascar, Gabon, Namibia, South Africa and Argentina; later, other collections were studied with material from Antilles, Vietnam, Central African Republic, Morocco, Congo, Thailand, Korea, China, Costa Rica, Brazil, Paraguay, etc.

We rapidly understood how essential it was to study type material deposited in different Museums; and in general collaboration was very good, but in some instances type material was not available on loan (National Natural History Museum, Budapest), leading to problems in the development of our study. A significant quantity of unavailable material is from Madagascar. We were fortunate as more than 4000 specimens from Madagascar were obtained from the Betsch and Paulian expedition team (Fernandez and Cleva 2010) and we managed to find specimens of all species unavailable on loan from Madagascar within these samples.

In this paper, we report on *Malgasodes curvisetus* (from the Madagascar collection at the MNHN) and *Malgasodes hungarorum* made available on loan from the Natural history Museum of Geneva, thus the totality of the species of this genus, with optic microscopy and SEM.

Identification of *Malgasodes curvisetus* was easy, due to several characteristics pointed out in the original description by Mahunka (2000), such as shape of interlamellar, lamellar, rostral, and notogastral setae; shape of sensillus and elevated interlamellar process.

We critically compared this genus with *Bovicarabodes* (Fernandez et al. 2013a) *Afticarabodes* (Fernandez et al. 2013b), *Cavaecarabodes* (Fernandez et al. 2014) and *Congocepheus* Balogh, 1958.

## Materials and methods

Specimens studied with light microscopy were macerated in lactic acid and observed in the same medium using the open-mount technique (cavity slide and cover slip) described by Grandjean (1949) and Krantz and Walter (2009). Drawings were made using an Olympus BHC compound microscope (Olympus, Rungis, France) equipped with a drawing tube. To aid observations, some specimens were stained with chlorazol black E (Coineau 1974). Specimens studied with the aid of scanning electron microscopy (SEM) were prepared as follows: specimens preserved in ethanol were carefully rinsed by sucking them into a Pasteur pipette several times, then transferring them for 2 hours to buffered glutaraldehyde (2.5%) in Sörensen phosphate buffer (pH 7.4; 0.1 m). After postfixation for 2 hours in buffered 2% OsO4 solution and rinsing in buffered solution, all specimens were dehydrated in a series of graded ethanols and dried in a critical point apparatus. Specimens were mounted on Al-stubs with double-sided sticky tape, after which they were gold-coated in a sputter apparatus (Alberti and Fernandez 1988; Alberti and Fernandez 1990a, 1990b; Alberti et al. 1991; Fernandez et al. 1991; Alberti et al. 1997; Alberti et al. 2007).

Measurements taken: total length (tip of rostrum to posterior edge of notogaster) and width (widest part of notogaster) in micrometres (μm). The description of leg chaetotaxy (SEM, standard, polarized and phase contrast microscopes) of *Malgasodes curvisetus* should be considered provisory as detailed study of leg chaetotaxy during ontogenetic development is necessary. Setal formulae of the legs include the number of solenidia (in parentheses); tarsal setal formulae include the famulus (*ε*).

### Morphological terminology

Morphological terms and abbreviations used are those developed by F. Grandjean (1928–1974) (cf. Travé and Vachon 1975), Norton and Pelletier (2009) and Fernandez et al. (2012, 2013a, 2013b).

Evans (1992) and Murley (1951) (In: Evans *op.cit.*) were followed for setal types and ornamentation of cuticular surfaces respectively. Aligned irregular furrows (*a.i.f*); bothridial ring (*bo.ri*); bothridial tooth (*bo.to*); circumgastric depression (*s.c*); elevated interlamellar process (*e.i.p*); notogastral anterior depression (*n.a.d*); posterior prodorsal depression (*p.p.d*); superior cornea of naso (*cso*).

## Taxonomy

### Genus Malgasodes

Taxon classificationAnimaliaOribatidaCarabodidae

Mahunka, 2000

**Figures 1–3.**
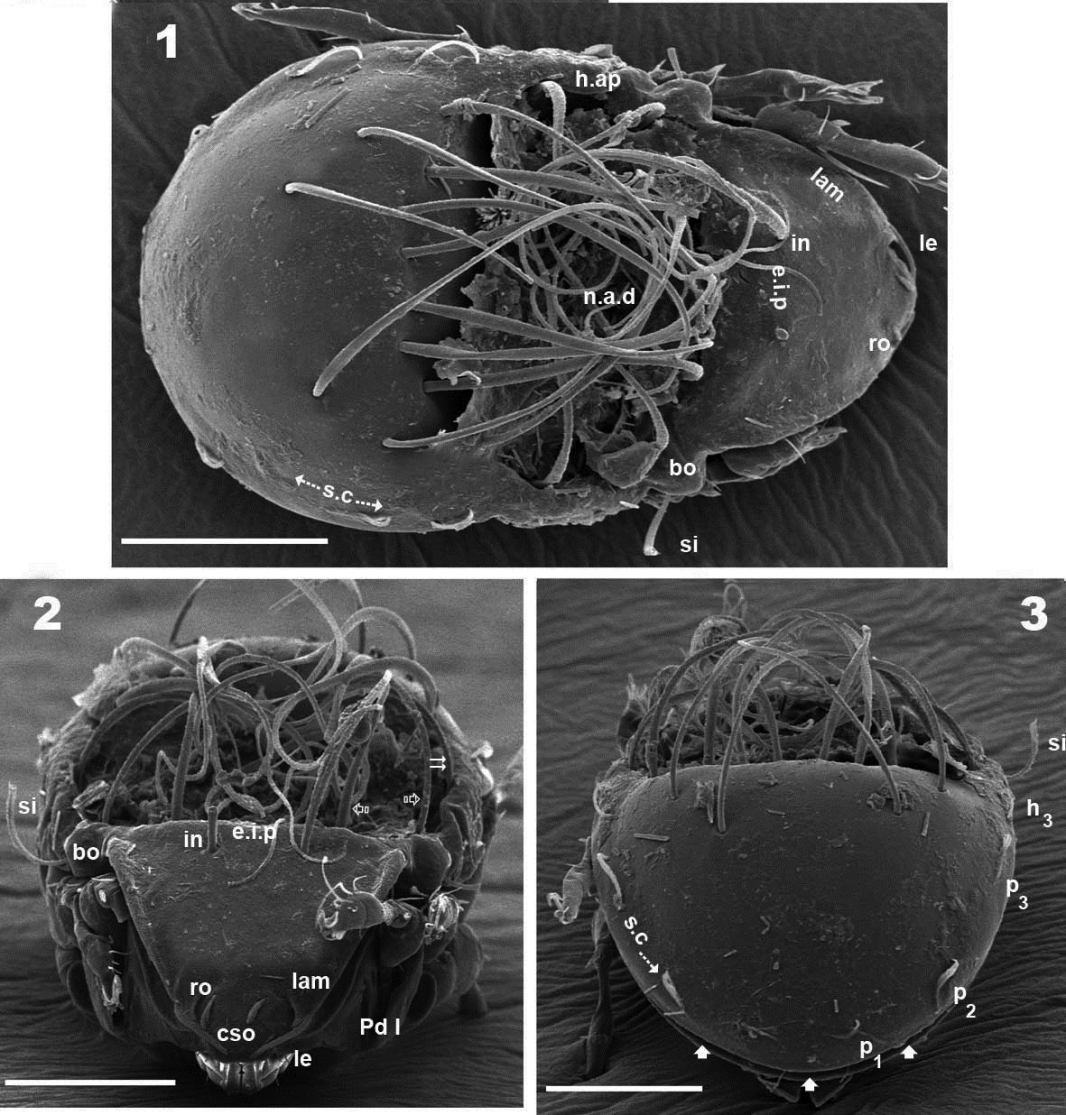
*Malgasodes curvisetus* Mahunka, 2000, adult. SEM observations. **1** dorsal view **2** frontal view **3** posterior view. Notes: Abbreviations: see “Material and methods”. Scale bar: **1–3** = 100 μm.

**Figures 4–5. F2:**
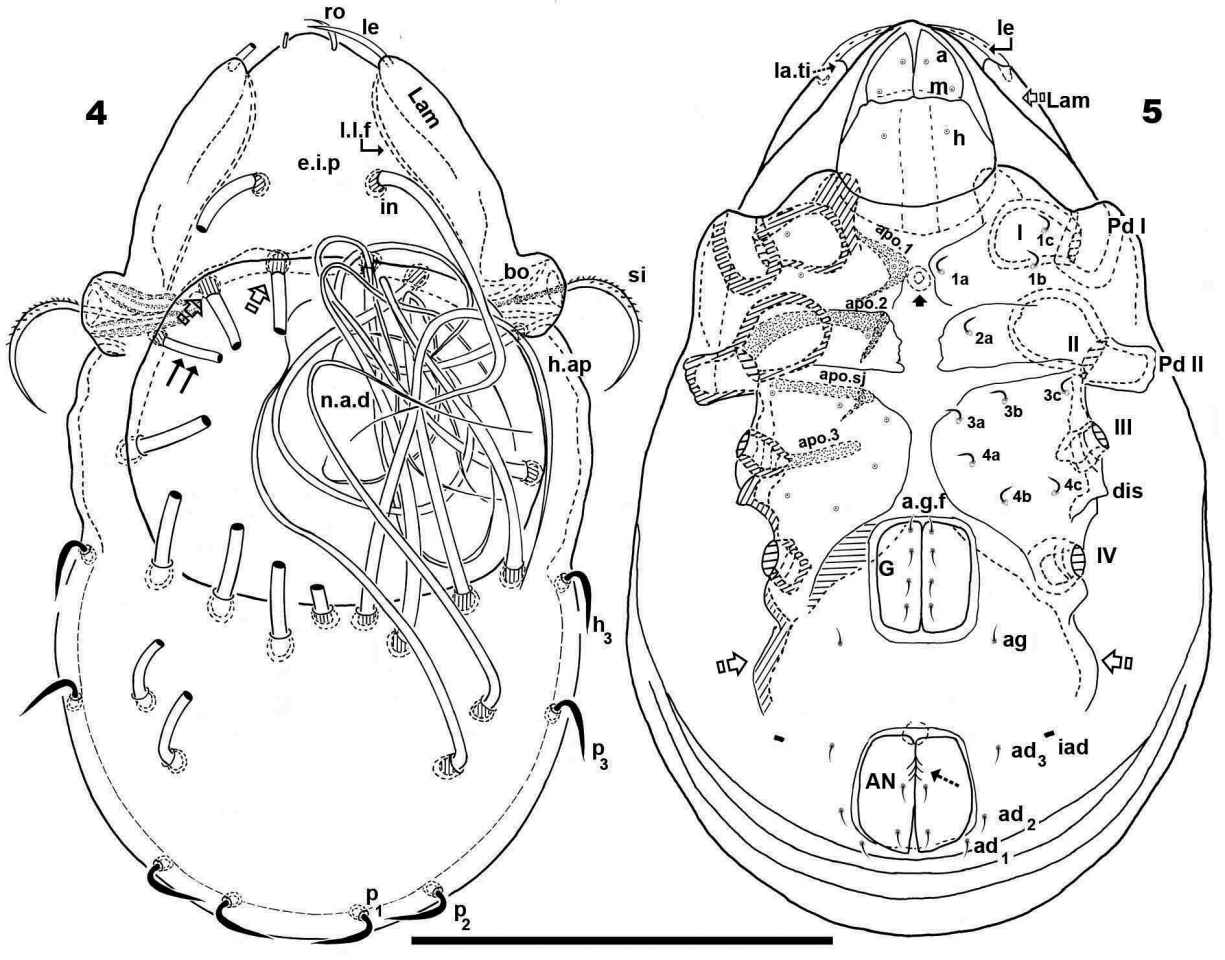
*Malgasodes curvisetus* Mahunka, 2000, adult. Optic observations. **4** dorsal view **5** ventral view. Notes: Abbreviations: see “Material and methods”. Scale bar **4, 5** = 250 μm.

**Figures 6–11. F3:**
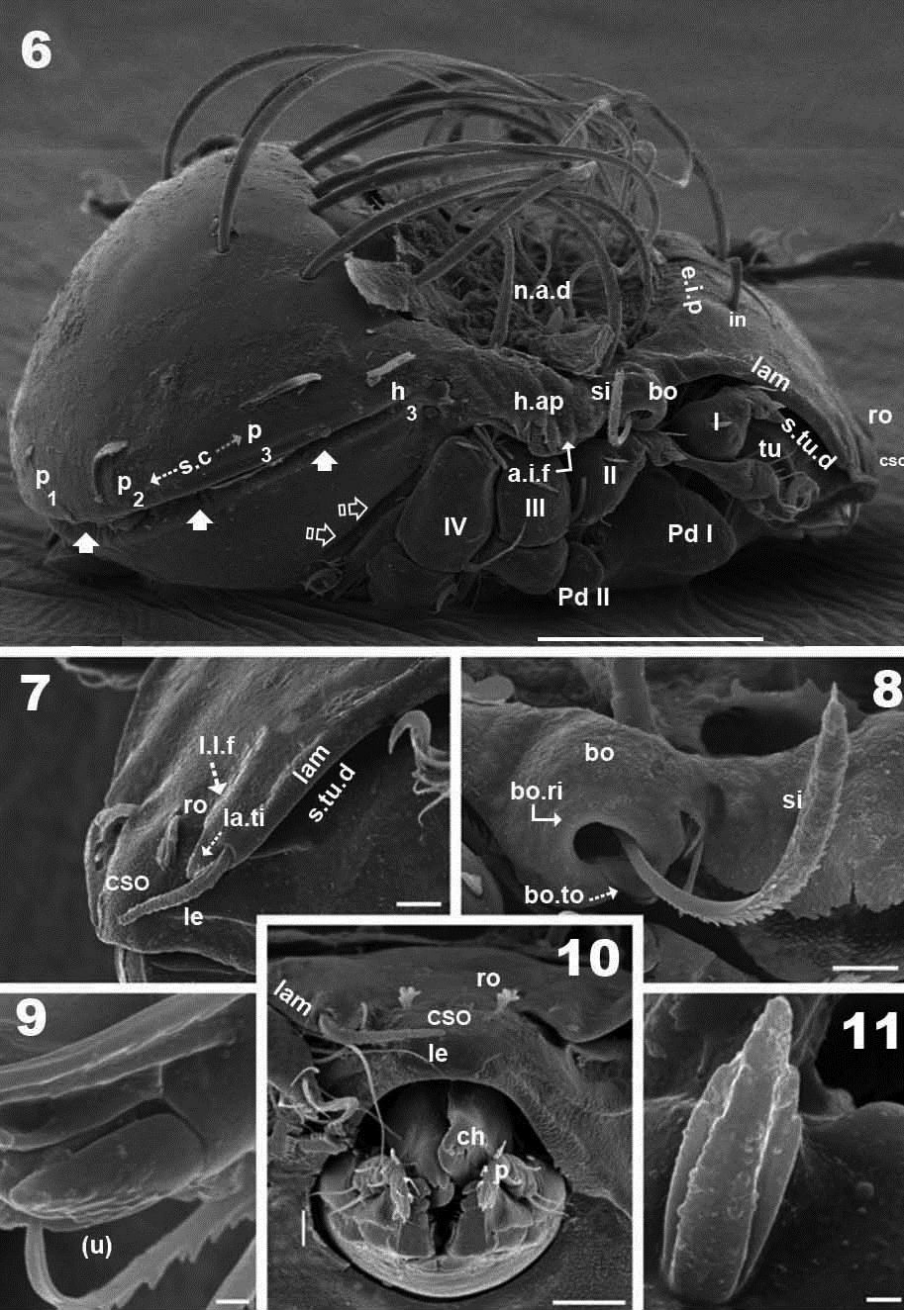
*Malgasodes curvisetus* Mahunka, 2000, adult. SEM observations. **6** lateral view **7** prodorsum, anterior part, lateral view **8** sensillus and anterior zone humeral apophysis **9** tarsus I, (*u*) seta, lateral view **10** rostrum and subcapitulum, frontal view **11** leg 1, segment l, genu. Notes: Abbreviations: see “Material and methods”. Scale bar: **6** = 100 μm; **7, 8, 10** = 10 μm; **10, 9, 11** = 1 μm.

Malgasodes curvisetus Mahunka (2000, 87 p) established the genus, with as type species (87–90 p, Figures 1–6).

#### Redescription.

**Diagnosis.** Prodorsum with elevated processes; without posterior prodorsal depression. Lamellae dorsolateral. Bothridia with smooth bothridial ring and bothridial tooth. Notogaster with conspicuous anterior notogastral depression, fourteen pairs of setae, four pairs of setae inside anterior notogastral depression; four pairs around notogastral depression; two pairs posterior and externally to depression; four pairs marginally to notogaster. Interlamellar and notogastral setae long, simple (except four marginal pairs). Supratutorial depression, Pedotecta I, II, discidium, humeral apophysis present. Notogastral border tectum not prolonged by a limbus, notogastral and ventral fig separated.

Epimeral chaetotaxy 3-1-3-3. Anterior genital furrow present. Four pairs of genital setae; aggenital setae posterior to genital opening. Three pairs of adanal setae; anal fig slightly tipped. Two pairs of anal setae.

### Type species

#### Malgasodes curvisetus

Taxon classificationAnimaliaOribatidaCarabodidae

Mahunka, 2000

Figures 1 [Fig F2] [Fig F3] [Fig F4] –17

##### Diagnosis.

**Redescription. *Shape*:** elongate ovoid.

***Integument*.** Prodorsum and notogaster smooth; only zone surrounding superior eye cornea, bothridium, and lateral body with small protuberances.

***Setation*.** *simple*, *small*: subcapitular, epimeric, genital, aggenital, anal, adanal; *simple*, *large*, *long sharply curving tip*: notogastral, surrounding, posterior to and inside notogastral anterior depression and *in* setae; *sausage-shaped*, *roughly-spiculate surface*: *le* setae; *phylliform*, *small*, elevated central zone: *ro* setae; *phylliform medium sized*: *h_3_*, *p_1_*, *p_2_*, *p_3_*.

***Prodorsum*.** Triangular to polyhedral. Intact elevated interlamellar process; *in* setae situated on elevated interlamellar process. Setae *in*, *ro*, *le* different shapes and lengths: *in*> *le*> *ro*; *in* setae directing posteriorly, exceeding the prodorsal margin. Rostral margin rounded. Shallow lamellar furrow discernible in the proximity of lamellar tip; lamellar tip triangular, small, shark tooth-like shape; superior cornea of naso, clearly visible; sensillus uncinate, curving upward. Notogastral anterior depression zone rectangular; posterior to this rectangular zone, an ovoid zone; dorsosejugal furrow hardy discernible, narrow, curving slightly backward. Circumgastric depression present, hardly discernible. Two pairs of notogastral setae situated posterior to notogastral anterior depression, extending forward. Four pairs inside notogastral anterior depression, with two pairs situated near dorsosejugal furrow, extending backward; four pairs bordering notogastral anterior depression, extending forward; four pairs marginally to notogaster, directing backward; three pairs of lyrifissures present, one pair at level of *h_3_* setae; one pair anterior to *p_3_* setae and *ips* between *p_1_* and *p_2_* setae.

Apodemes *apo.1*, *apo.2*, *apo.sj* and *apo.3* clearly visible; at level *apo.1*, in medial zone, rounded structure visible. Four pairs of linearly placed genital setae. Aggenital setae posterolaterally, three pairs of adanal setae; small, elongate bean-shaped lyrifissures *iad.* Two pairs of anal setae. Smooth surface between genital and anal openings.

##### Material examined.

Scanning Electron Microscopy and optic observation: 6 specimens; Madagascar R.C.P 2010. «Région Fort-Dauphin Madagascar Sud-est.» «R.C.P 2010-Piste de Ste Luce-Forêt littorale-Altitude 10 mètres» 9-12-1971. Coll. J-M.BETSCH. Three specimens deposited in Muséum National d’Histoire Naturelle, (MNHN) Paris, France.

##### Adult description.

**Female. *Measurements*:** 372 μm (400–351) × 260 μm (239–281) (three specimens). All specimens were female.

***Shape*:** elongate ovoid (Figures 1, 4, 5).

***Colour*:** yellow to light brown; slightly shiny, when observed in reflected light.

***Cerotegument*.** Present in the notogastral anterior depression (*n.a.d*), retained by the setae and on humeral apophysis. On other body parts and legs nonexistent or disappeared during observation in lactic acid; similar in SEM observations (Figures 1, 6).

***Integument*.** Prodorsum: elevated interlamellar process (*e.i.p*) smooth (Figures 1, 2, 6); zone surrounding *CSO*, *bo*, and lateral body zone with small protuberances (Figures 7, 8, 10). Notogaster: smooth (Figures 1, 3, 6).

**Figures 12–15. F4:**
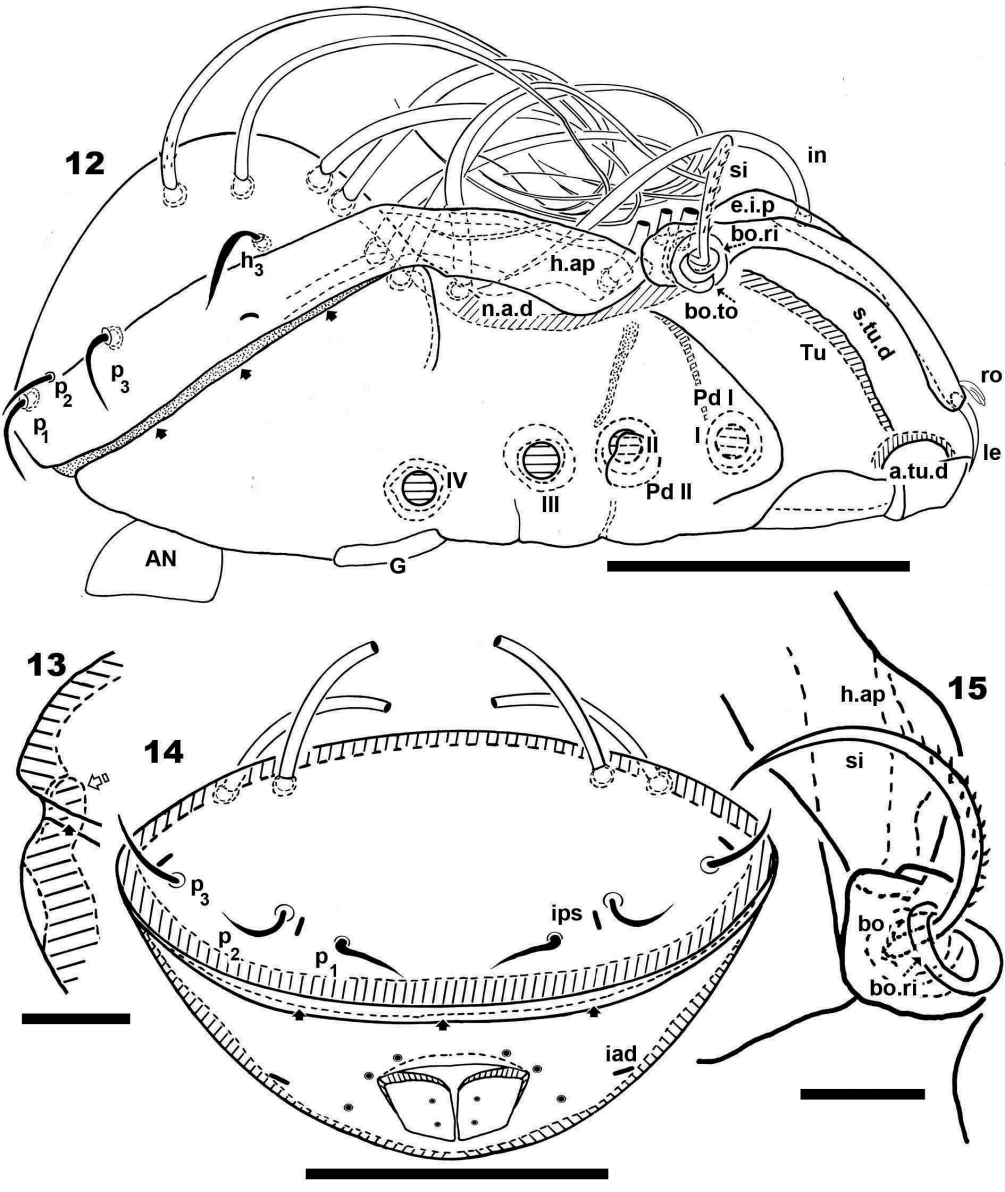
*Malgasodes curvisetus* Mahunka, 2000, adult. Optic observations. **1** dorsal view **2** ventral view **3** frontal view. Notes: Abbreviations: see “Material and methods”. Scale bar: **12** = 250 μm; **13** = 2 μm; **14** = 200 μm; **15** = 4 μm.

***Setation*** (legs not included). Five types: 1) *simple*, *small*: subcapitular, epimeric, genital, aggenital, anal, adanal (Figure 5); 2) *simple*, *large*, *long sharply curving tip*: notogastral setae around, behind and inside *n.a.d*, and *in* setae (Figures 1, 2, 3, 4, 6); 3) *sausage-shaped*, *roughly-spiculate surface*: *le* (Figure 7); 4) *phylliform*, *small*, with elevated central zone, delimited on both sides by longitudinal depression; central elevated zone with longitudinal furrow in central zone giving a particular aspect in frontal view (Figure 10): *ro* setae (Figures 2, 7, 17); 5) *phylliform*, *medium size*: notogastral *h_3_*, *p_1_*, *p_2_*, *p_3_* (Figures 1, 3, 4, 6, 12, 14).

**Figures 16–19.**
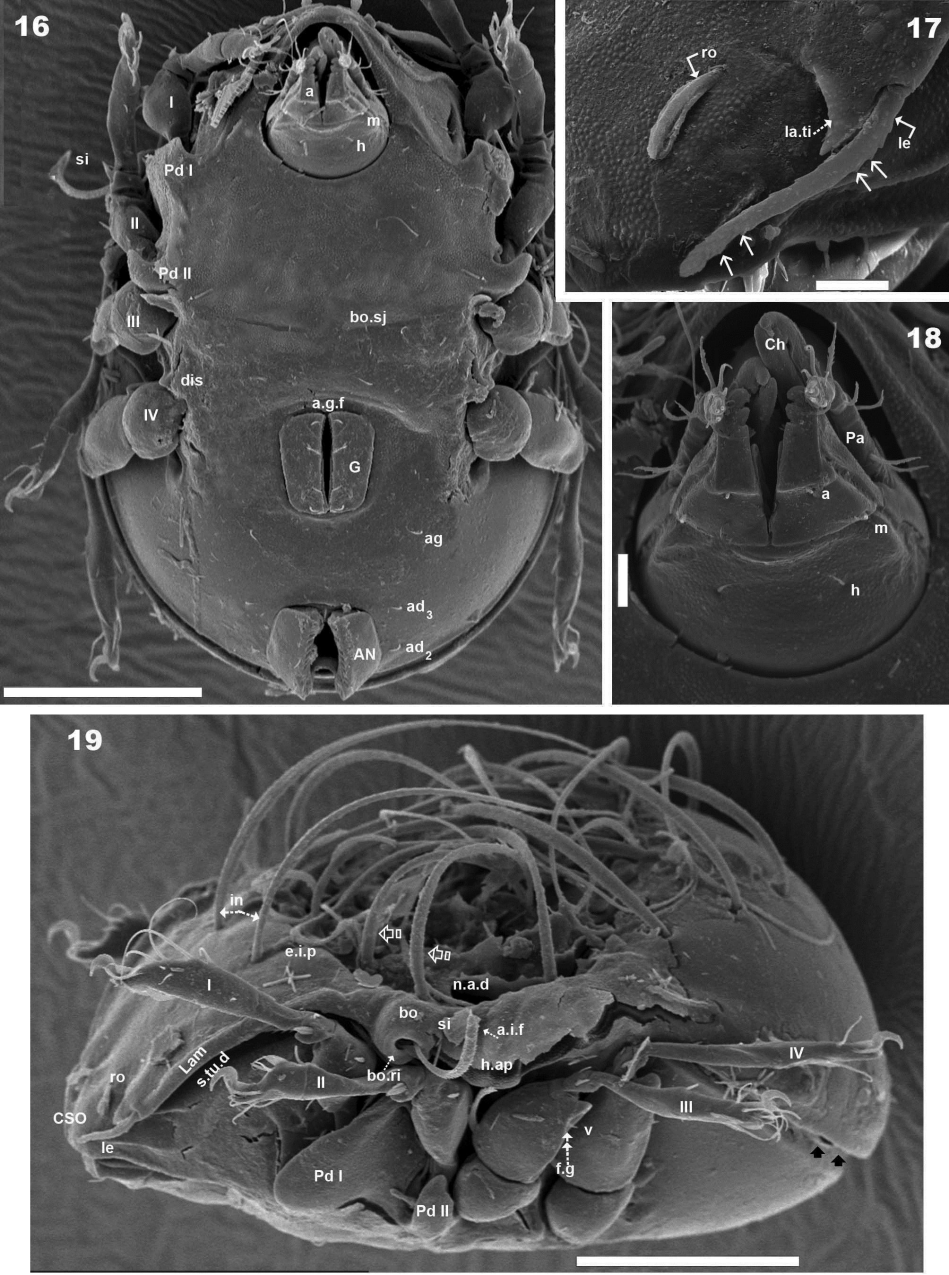
*Malgasodes curvisetus* Mahunka, 2000, adult. SEM observations. **16** ventral view **17** lamellae and lamellar tip **18** subcapitulum **19** lateral view, rotated. Abbreviations: see “Material and methods”. Scale bar: **16, 19** = 100 μm; **17, 18** = 10 μm.

**Figures 20–23.**
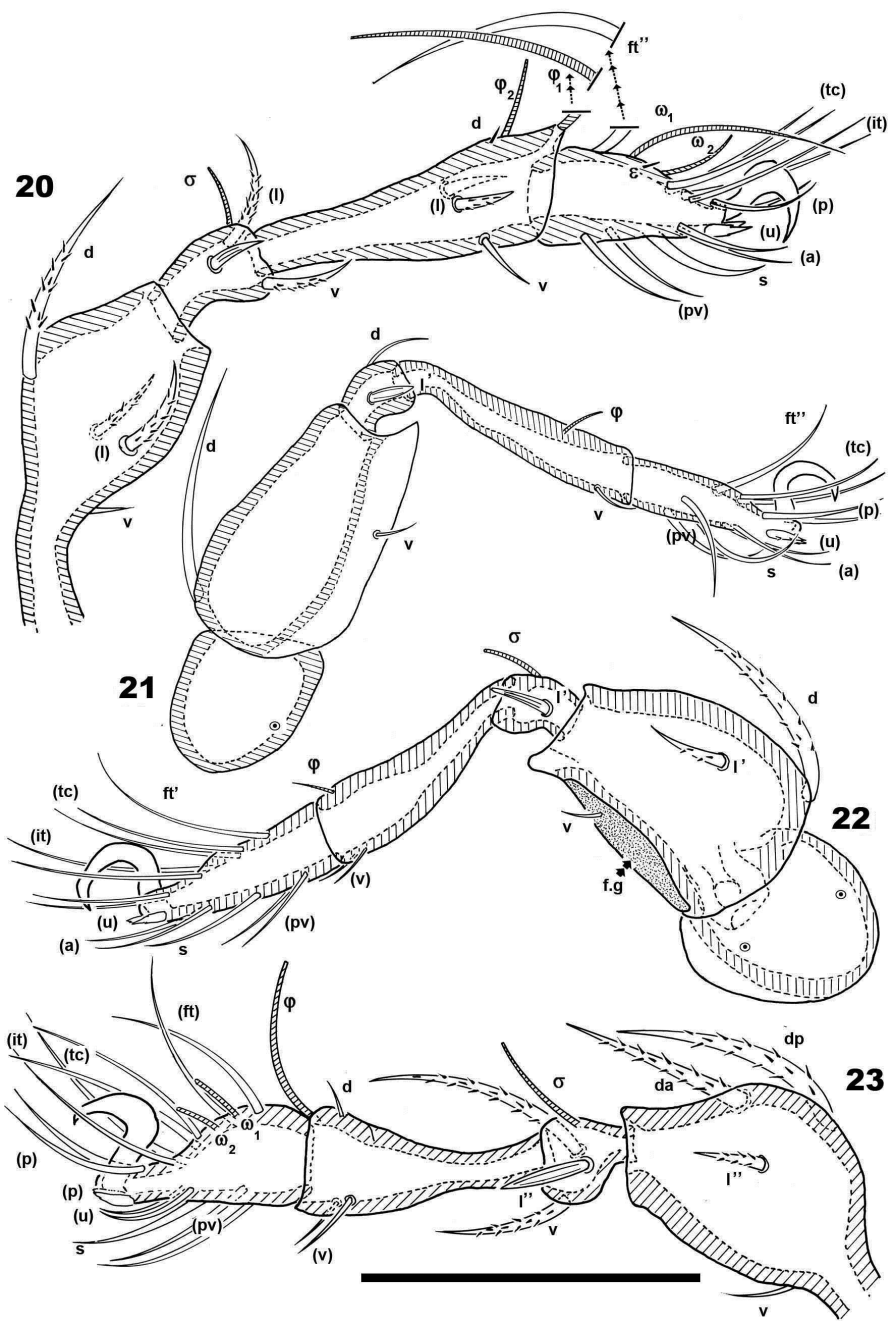
*Malgasodes curvisetus* Mahunka, 2000, adult. Legs. Optic observations **20** leg I antiaxial view **21** leg IV, antiaxial view **22** leg III, view **23** leg II, antiaxial view. Notes: Abbreviations: see “Material and methods”. Scale bar: **20–23** = 100 μm.

***Prodorsum*.** Triangular to slightly polyhedral (dorsal view) (Figures 1, 4); triangular in lateral and frontal view (Figures 2, 6, 12). Entire elevated interlamellar process (*e.i.p*) (Figure 2) situated at lower level than superior notogastral limit (Figures 2, 3, 6, 12).

Posterior prodorsal depression (*p.p.d*) absent (Figures 1, 6). Setae *in* situated on *e.i.p* (Figures 1, 2, 4, 6, 12), similar characteristics to long simple notogastral setae situated near and inside *n.a.d*; in all cases the *in* setae directing backward and entangled with cited notogastral setae (Figures 1, 2, 4, 6, 12).

Three pairs of prodorsal setae (*in*, *ro*, *le*) very different in shape and length (see Setation): *in*> *le*> *ro*; *ro* setae small, inserted posteriorly to *le* insertion, directing forward (Figures 1, 2, 4, 7, 10, 12, 14); *in* setae inserted on *e.i.p* antiaxially to medial plane and slightly externally to *ro* insertion level; posteriorly directed, far exceeding the prodorsal margin extending to *n.a.d* (Figures 1, 2, 4, 12, 19); *le* setae (Figures 1, 2, 4, 7, 10, 12, 14) laterally situated on lamellar apical zone but far from lamellar tip (*la.ti*), directing forward with tips converging to medial plane; *le* setae always found on prodorsal surface. Rostral margin rounded (Figures 1, 2, 4, 7, 10). Lamellae running laterally; shallow lamellar furrow (*l.l.f*) only discernible in proximity of *la.ti* (Figure 17); *la.ti* more or less triangular, small, sharply tipped (Figure 17). In frontal view (Figures 2, 10) the superior cornea of naso (*cso*) is clearly visible as a convex elevation situated anterior to the *ro* setal insertion; upward curving sensillus (*si*) uncinate with small barbs (Figure 2, 4, 6, 8, 12, 15).

Bothridium (*bo*), round–ovoid; bothridial ring (*bo.ri*) smooth, with bothridial tooth (*bo.to*), clearly visible (Figure 8, 12, 15).

***Notogaster*.** In dorsal view, zone of *n.a.d* rectangular; posterior to this zone, ovoid (Figures 1, 4); in lateral view anterior part (zone of *n.a.d*) depressed, concave whilst rest of notogaster convex (Figure 6, 12); *d.sj* hardly discernible, narrow, curving slightly backward.

Notogaster smooth (Figures 1, 3, 6, 12). Anterior notogastral depression (*n.a.d*) ovoid, conspicuous, large, extending forward (Figures 1, 4, 6, 12).

Circumgastric depression (*s.c*) present, hardly discernible (Figure 1, 3), situated at notogastral lateral setal insertion level (*p_1_*, *p_2_*, *p_3_*, *h_3_*).

Fourteen pairs of notogastral setae, two pairs situated posterior to *n.a.d*, extending forward, exceeded *d.sj*; four pairs inside *n.a.d*, of which two pairs situated near *d.sj* extending backward (indicated in Figure 4, 19 with X); two pairs situated far from *d.sj*, extending forward, exceeding *d.sj* (one of them indicated in Figure 4, 19 with J); four pairs situated marginally to *n.a.d*, extending forward, exceeding *d.sj* and four pairs, situated marginally to notogaster, directing backward, these pairs we named *h_3_*, *p_1_*, *p_2_*, *p_3_* (Figures 1, 3, 4, 6, 12, 14, 19).

Lyrifissures difficult to observe; three pairs present, first pair situated at level of *h_3_* setae (Figure 12), another situated anterior to *p_3_* setae and *ips* situated between *p_1_* and *p_2_*. Lyrifissures not visible in SEM, only under optic observation (Figures 12, 14).

Humeral apophysis (*h.ap*) clearly discernible principally in lateral view (Figures 1, 4, 6, 8, 12, 19), antiaxially aligned irregular furrows (*a.i.f*) present, permitting concealment of *si* during protection mechanism deployment (Figures 6, 19, indicated by arrows). Cerotegumental layer often covering *h.ap* (Figures 6, 8, 19). The *h.p* and the posterior bothridial zone in the junction zone between both structures, posterior bothridial tip overlapping *h.ap* anterior margin (Figure 6, 12, 19). Tectum border remarkable, not prolonged by a limbus, but by transverse cuticular structure (Figure 13 indicated by X) delimiting a space between notogastral and ventral shield (indicated by arrows· Figures 6; 12, 13, 19).

***Lateral region*.** Tutorium (*tu*) clearly visible as a strongly curving cuticular thickening. Between lamellae and tutorium a deep supratutorial depression (*s.tu.d*) running parallel to both structures (Figures 6, 7, 12, 19). Bothridia cup-shaped with smooth bothridial ring (*bo. ri*); *bo.ri* incomplete with bothridial tooth (*bo.to*) clearly visible (Figures 8, 12, 15, 19); sensillus (*si*) uncinate with small barbs, curving upward (Figures 8, 15) tip usually pointed, more coarsely barbed on lateral edge.

Lamellae with *la.ti*, short, “shark tooth-like” (Figures 7, 17); *le* setae sausage-shaped (Figure 17), roughly-spiculate surface, clearly visible (Figure 17 indicated by arrowh); *ro* phylliform, with rounded elevated central zone, delimited on each side by, depressed longitudinal furrow (Figure 17); *n.a.d* well discernible due to transparency (Figure 12); two pairs of setae (probably *c_1_*, *c_2_*) situated in the anterior part of *n.a.d* and close to *d.sj*, (Figures 4, 6, 12, 19 indicated by X), observating *d.sj*, complicated by presence of cerotegument and setae, requiring observation from different angles. Two other pairs of setae situated inside *n.a.d*; first pair laterally and close to two anterior setae, at level of bothridia (Figures 4, 12, 19 indicated byJ); the second pair hardly discernible, easily confused with other setae, situated marginally to *n.a.d*. Lateral view permitting clear understanding, of different setae directions and perceiving the complexity found at *n.a.d* level (Figures 6, 12, 19).

Pedotectum I, prominent extended lamina. Pedotectum II, small polyhedral lamina, rounded edges. Humeral apophysis more or less triangular; basally slightly convex, immediately becoming concave; posterior bothridial zone overlapping anterior tip (Figure 12); aligned irregular furrows (*a.i.f*) delimited by rod-like cuticular structures (Figures 6, 19, indicated by arrow), crossing *h.ap*.

SEM observations made from two different lateral angles (Figures 6, 19) in order to clarify the relative position, shape and disposition of different prodorsal and notogastral structures and setae. In order to show positioning of legs during “legs folding process’ specimens are shown with legs in place and alternatively with distended legs. Only one lyrifissure visible at level of *h_3_* setae Discidium not discernible. Only one depression, situated behind leg IV, used conceal tibia and tarsus (paraxial side) (Figure 6 indicated by X) during leg folding process (see Fernandez et al. 2013b).

***Posterior view*.** Posterior view (Figure 14) clarifying position of marginal setae *p_1_*, *p_2_*, *p_3_*, lyrifissures and the two pairs of notogastral setae situated posterior to *n.a.d*, unique pairs visible in posterior view. Lyrifissure *ips* can be uniquely identified on account of placement between setae *p_1_* and *p_2_*; the other, probably *ih*, situated between *p_3_* and *h_3_.*

**Table 1.** *Malgasodes curvisetus* Setae and Solenidia.

Leg I	Femur	Genu	Tibia	Tarsus	Claw
Seta	*d*, *(l)*, *v*	*(l)*, *v*	*d*, *(l)*, *v*	*ft*”, *ε*, *(tc)*, *(it)*, *(p)*, *(u)*, *(a)*, *s*, *(pv)*	1
Solenidia	--------------	σ	-φ_1_, φ_2_	-----ω_1_, ω_2 -_	
Leg II					
Seta	*da*, *dp*, *l*”, *v*	*(l)*, *v*	*d(v)*	*(pv)*, *s*, *(a)*, *(u)*, *(p)*, *(it)*, *(tc)*, *(ft)*	1
Solenidia	-------------	-σ-	-φ-	ω_1_, ω_2_	
Leg III					
Seta	*d*, *l*’, *v*	*l*’	*(v)*	*ft*’, *(pv)*, *s*, *(a)*, *(u)*, *(tc)*, *(p)*, *(it)*	1
Solenidia	---------------	-σ-	-φ-	------------0--------------	
Leg IV					
Seta	*d*, *v*	*d*, *l*’	*v*	*ft*”, *(a)*, *(pv)*, *(tc)*, *(p)*, *(u)*, *s*	1
Solenidia	---------------	---0	--φ-	------------0------------------	

In this position the zone between ventral and notogastral figs is easily discernible, separated due to particular tectal border (indicated in Figure 14 by ·).

***Ventral region*.** Subcapitulum with three pairs of setae (*a*, *m*, *h*) clearly visible; insertion zone *m* and *a* setae simple, smooth (Figures 16, 18). Epimera hardly discernible; only *bo.sj* clearly visible as shallow furrow crossing the medial plane (Figure 16).

Apodemes *apo.1*, *apo.2*, *apo.sj* and *apo.3* clearly visible (Figure 5). In median zone, at level of *apo.1*, rounded more sclerotized structure visible (in optic observation), but smooth under SEM observation. Epimeral chaetotaxy 3-1-3-3.

Genital fig more or less similar in size to anal fig (Figure 5); anal fig small, sharply tipped (Figure 16); paraxial border of anal figs with small teeth (Figure 5, indicated by 5) on anterior third.

Anterior to genital fig a furrow (*a.g.f*) clearly visible. Four pairs of linear genital setae. Aggenital setae posterolaterally, posterior opening of genital border (Figure 5) situated at same level or slightly antiaxially to *ad _3_* setal insertion. Three pairs of adanal setae; small elongate bean-shaped lyrifissures *iad* clearly visible, situated antiaxially and far from *ad_3_*. Two pairs of anal setae. Smooth surface between and lateral to genital and anal openings (Figure 16); depression at level of leg IV (involved in leg folding process. See lateral region). Short, curving, cuticular thickening behind acetabulum IV (Figure 5 indicated by X).

***Legs*.** Legs presenting all characteristics observed in other Carabodidae, but lateral setae of genua are particular (Figure 11). The (*u*) pair of all tarsi are without barbs but rugous, particular (Figure 9).

All structures related to leg folding are present (Fernandez et al. 2013a) and clearly visible (Figures 6, 12, 16, 19).

Setal formulae I (1-4-3-4-15-1) (1-2-2); II (1-4-3-3-15-1) (1-1-2); III (2-3-1-2-14-1) (1-1-0); IV (1-2-2-1-12-1) (0-1-0).

##### Remarks.

Anterior part of *n.a.d* extending to *d.sj*, together with notogastral setae situated in this zone preventing clear observation. The *e.i.p*. is an elevated process situated not far from *d.sj*. The posterior part of *e.i.p* descending steeply at *d.sj* level. We consider *p.p.d* to be absent.

Two pairs of setae situated close to *d.sj* are probably *c_1_*,and *c_2_* setae, but without immature stases it was impossible to establish with certainty.

Border of tectum in *Malgasodes* is unlike all others; in that it is not prolonged by a limbus, and that a space exists between the ventral fig and the notogaster.

Various inclinations in lateral view are provided to permit better understanding of the disposition of notogastral setae and the steeply descending posterior part of *e.i.p* towards *d.sj*.

### Complementary description

#### Malgasodes hungarorum

Taxon classificationAnimaliaOribatidaCarabodidae

Mahunka, 2010

Figures 24–27

##### Remarks.

Only one bleached specimen was available for study. The condition of this material was poor, for this reason a decision was made to add only characteristics and figures considered inadequate in the original description by Mahunka (2000). The ventral zone (text and figure) contained adequate information; some omitted aspects will be addressed in text. In contrast the dorsal and lateral descriptions and figures require redescription and new figures.

##### Diagnosis.

Ovoid. Notogaster, prodorsum smooth; anterior zone *lam*, lateral body zone, small protuberances

Rostrum rounded to polyhedral. Complete elevated interlamellar process. Posterior prodorsal depression not present; *in* setae simple, medium length, situated on elevated interlamellar process, directing posteriorly; *ro* small, phylliform, slightly barbate, directing forward; *le* sausage-shaped, slightly barbate, directing forward; *in*> *le*> *ro.*

Lamellae laterally; shallow lamellar furrow well discernible dorsally; lamellar tip rounded to polyhedral. Superior cornea of naso clearly visible; sensillus uncinate, small barbs, curving upward; bothridial ring smooth, bothridial tooth present. Anterior notogastral depression well discernible, conspicuous; ovoid-shaped anterior zone, polyhedral posterior border. Circumgastric depression present, hardly discernible.

Fourteen pairs of notogastral setae, two pairs outside posterior notogastral anterior depression directing forward; four pairs inside notogastral anterior depression, three pairs near dorso-sejugal furrow, fourth pair distant; four pairs marginally to notogastral anterior depression; four pairs marginally to notogaster; two pairs lyrifissures present. Humeral apophysis easily discernible.

Tutorium strongly curving cuticular thickening; supratutorial depression clearly visible.

Pedotectum I, prominent extended lamina. Pedotectum II, small, polyhedral; two lyrifissures present; discidium not discernible; sejugal zone depressed; semicircular ridges at acetabulum IV level. Epimeral setae simple, fine; epimeric formulae 3-1-3-3; *1c* small. Aggenital furrow present; anogenital region with ribs and crest; genital setae 4 pairs; aggenital 1 pair; anal 2 pairs; aggenital 3 pairs; *iad* present, situated far from anal opening.

##### Material examined.

**Paratype:** Madagascar, Tomasina Province, Mananara Nord Biosphere Reserve and National Park, Lowland rainforest, NW slope Behafotra Hill; 250–300 m alt. 16°27. 1–3'S, 49°47.6'E.14–15 August 1988. N° 9877. Leg. T. Pócs. Deposited in the Museum of Natural History, Geneva (MNHG).

***Measurements*:** 301 μm × 198 μm.

***Shape*:** ovoid (Figure 25).

**Figures 24–27.**
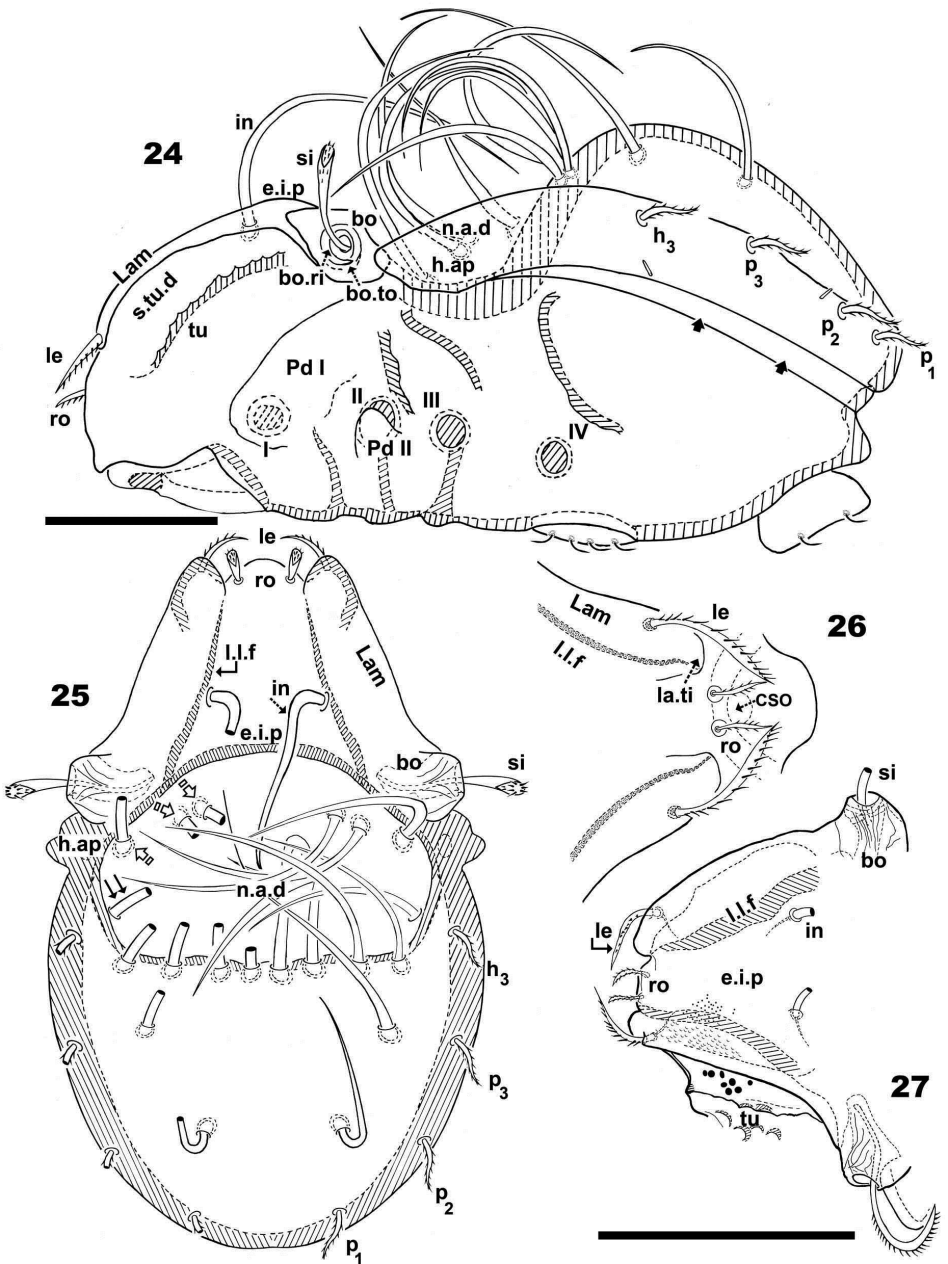
*Malgasodes hungarorum* Mahunka, 2000, adult. Optic observations. **24** lateral, slightly inclined view **25** dorsal view **26** prodorsum anterior part, dorsal view, inclined anteroposterior **27** prodorsum dorsal, inclined laterally. Abbreviations: see “Material and methods”. Scale bar: **24–25** = 60 μm; **26, 27** = 55 μm.

***Colour*:** Yellow to light brown; slightly shiny, when observed in reflected light.

***Cerotegument*.** Not observed.

***Integument*.** Notogaster and prodorsum smooth; anterior zone of *lam* and lateral body zone with small protuberances (Figures 24, 25, 27).

***Prodorsum*.** Triangular to slightly polyhedral (dorsal view) (Figures 25, 27); curving in lateral view (Figure 24). Complete *e.i.p* (Figures 24, 25).

Posterior prodorsal depression (*p.p.d*) absent (Figures 24, 25). Setae *in*, medium length, situated on *e.i.p*, similar to long simple notogastral setae situated adjacent to and inside *n.a.d*, in all cases *in* setae directing backward and entangled with the cited notogastral setae (Figures 24, 25 ).

Three pairs of prodorsal setae (*in*, *ro*, *le*) (Figures 24–27), very different in shape and length: *in*> *le*> *ro*; *ro* setae small, phylliform, slightly barbate, directing forward; *in* setae inserted on *e.i.p* antiaxially to medial plane and slightly externally to *ro* insertion level, close to *l.l.f*; directing posteriorly, exceeding the prodorsal margin, extendingto *n.a.d*; *le* setae sausage-shaped, slightly barbate, observation difficult (depending on position/angle of observation), laterally situated on lamellar apical zone, but far from *la.ti*, directing forward. Rostral margin rounded to polyhedral (Figures 25, 26).

Lamellae running laterally; *l.l.f* well discernible (Figures 25, 26, 27); *la.ti* rounded to polyhedral. Superior cornea of naso (*cso*) clearly visible as convex elevation situated anterior to *ro* setae insertion level (Figures 26); upward curving sensillus (*si*), uncinate with small barbs (Figure 27).

Round-ovoid *Bo* with longitudinal ridges; *bo.ri* smooth, clearly visible *bo.to* (Figure 24).

***Notogaster*.** Dorsal view: ovoid with polyhedral anterior zone of *h.ap.* (Figure 25). Lateral view, anterior part (zone of *n.a.d*) depressed, concave and rest convex (Figure 24); *h.ap* prominent expansion, large rounded tip (Figures 26, 27).

Notogaster smooth (Figure 25). Anterior notogastral depression (*n.a.d*) easily discernible, conspicuous, large, extending forward, ovoid anterior zone, polyhedral posterior border (Figure 25).

Circumgastric depression (*s.c*) present, hardly discernible, situated paraxially to lateral notogastral setal insertion (*p_1_*, *p_2_*, *p_3_*, *h_3_*).

Fourteen pairs of notogastral setae, two pairs situated posterior to *n.a.d*, one far from posterior border of *n.a.d*; both pairs directing forward, not exceeding *d.sj*; four pairs inside *n.a.d*, three pairs situated near *d.sj* extending backward or laterally (indicated in Figure 25 with X); fourth pair situated far from *d.sj*, extending forward or laterally, not exceeding *d.sj* (indicated in Figure 25 withJ); four pairs situated marginally to *n.a.d*, extending forward; four pairs, situated marginally to notogaster, directing backward, these four pairs are possibly named as *h_3_*, *p_1_*, *p_2_*, *p_3_* (Figure 25).

Lyrifissures difficult to observe; two pairs present, first pair situated at level of *h_3_* setae, the other situated between *p_3_* and *p_2_* (Figure 24).

Humeral apophysis (*h.ap*) clearly discernible (Figures 24, 25). Posterior bothridial zone overlapping anterior tip of *h.ap* (Figures 24, 25).

Remarkable border tectum, not prolonged by a limbus, space existing between the notogastral and ventral figs (Figures 24, indicated by arrows ·).

***Lateral region*.** Tutorium (*tu*) clearly visible as a strongly curving cuticular thickening. Between lamellae and tutorium, deep supratutorial depression (*s.tu.d*) running parallel to both structures (Figure 24). Bothridia cup-shaped with smooth bothridial ring (*bo. ri*); *bo.ri* incomplete with *bo.to* clearly visible (Figures 24); *si* uncinate with small barbs, curving upward (Figure 27, pointed tip).

Lamellae with *la.ti*, round-polyhedral (Figures 26, 27); *le* setae sausage-shaped, rough-barbate surface, viewing angle slightly changing observation (see both sides of drawing Figure 27); *ro* small, philliform, barbate (Figures 26, 27); *n.a.d* clearly discernible; *d.sj* hardly visible. Lateral view greatly assists in understanding of different setae directions, and perceiving complexity found at *n.a.d* level (Figure 24).

Pedotectum I, prominent extended lamina, rounded apex. Pedotectum II, small polyhedral lamina, rounded edges. Humeral apophysis triangular; basally slightly convex and immediately concave; anterior tip overlapping posterior bothridial zone (Figure 24).

Only two pairs of lyrifissures visible at level of *h_3_* setae and between *p_3_* and *p_2_*. Discidium not discernible. Sejugal zone depressed. Semicircular ridges at level of acetabulum IV (Figure 24).

***Ventral region*.** Well described by Mahunka (2000: page 89 and Figure 6) only adding: the *a.g.d* is clearly visible.

### The comparison

*Bovicarabodes* Fernandez, Theron & Rollard, 2013a, *Afticarabodes* Fernandez, Theron & Rollard, 2013b, *Cavaecarabodes* Fernandez, Theron & Rollard, 2013c, *Congocepheus* Balogh, 1958, *Malgasodes* Mahunka, 2000.

### Introduction

Many years of study and previous publications lead us to the point where we are able to compare these genera. Several new collections with large numbers of specimens have become available, but it will take several years to describe this new material. A decision was made to first complete the comparison of the cited genera before commencing further descriptions.

In previous publications, we described *Bovicarabodes*, *Afticarabodes*, *Cavaecarabodes*, and studied and redescribed most of the species of *Congocepheus*. All genera present several characteristics in common such as: notogastral anterior depression, posterior prodorsal depression, superior cornea of naso, elevated interlamellar process, notogastral setae situated around the notogastral anterior depression; humeral apophysis; anterior genital furrow. We observed two evolutive phenomena: regression and neotrichy in *Cavaecarabodes anouchkae* and *Congocepheus germani*, respectively.

*Malgasodes* presents particular characteristics and some characters in common with genera mentioned above. Firstly we explain the situation of *Malgasodes*, after which we complete the comparison.

*Malgasodes* consists of only two species (Subias 2013) *Malgasodes curvisetus* Mahunka, 2000 and *Malgasodes hungarorum* Mahunka, 2000, both from Madagascar, described in the same with *Malgasodes curvisetus* as type species (Holotype and paratype deposited in Hungarian Natural History Museum, Budapest) and *Malgasodes hungarorum* (Holotype and 4 paratypes deposited in the Hungarian Natural History Museum, Budapest, and 1 paratype in the Musée d’Histoire Naturele, Genève (MHNG)).

We studied specimens of both *Malgasodes hungarorum* (paratype) on loan from MHNG and *Malgasodes curviseta* unavailable from the Hungarian Natural History Museum, Budapest, but fortunately found in the Madagascar collection of Professor J-M Betsch (between 1968 and 1973), deposited in Museum National d’Histoire Naturelle, Paris, France (MNHN).

In the original description of *Malgasodes*, Mahunka (2000: 87) indicated: “Notogaster with a wide deep, roughly semicircular, hollow anteriorly, well framed laterally and posteriorly”. “Fourteen pairs of notogastral setae: ten pairs long medially directed, recurved and four pairs short, phylliform, in marginal position. Of these 14 pairs, eight pairs arise around hollow, two pairs arise posteriorly and remaing four pairs present in posteromarginal position”.

However, in Figure 1 (page 88) it is clear that four pairs of the eight indicated arise from and originate inside the *n.a.d* (“hollow” *sensu* Mahunka) and are not all situated around the *n.a.d.* In our studies we confirmed that four pairs are inside *n.a.d* (see redescription of *Malgasodes cuvisetus* and *Malgasodes hungarorum*).

In the comparison with other genera Mahunka indicated “Its allies are in the genre *Congocepheus* Balogh, 1958 and *Baloghodes* Mahunka, 1986. However, *Congocepheus* is clearly distinguished from *Malgasodes* by the form of the ventral structure, mainly the ventral fig and the position of the lyrifissures *iad.* Species of the genus *Baloghodes* are characterized by the absence of the anteromedian hollow and covered lateral margin”.

Mahunka only considerered the ventral characteristics in *Congocepheus* but there are other more important characteristics (see Table 2).

**Table 2.** Comparison between *Bovicarabodes*, *Afticarabodes*, *Cavaecarabodes*, *Congocepheus*, *Malgasodes*.

	*Bovicarabodes*	*Afticarabodes*	*Cavaecarabodes*	*Congocepheus*	*Malgasodes*
*n.a.d*	present	present	present	present	present
*p.p.d*	present	present	present	present	absent
Notogastral setae outside *n.a.d*	14 pairs	14 pairs	14 pairs	14 pairs	10 pairs
Notogastral setae inside *n.a.d*	0	0	0	0	4 pairs
Orthotrichy bideficient	present	present	present*^3^*	present*^1^*	present
Notogastral setae identify	All	All	All	All	*p_1_*, *p_2_*, *p_3_*, *h_3_*
Notogastral setae setiform	0	0	0	1-2 pairs*^2^*	10 pairs
*e.i.p*	present	present	present	present	present
Prodorsum	Paired horns and two cavities	Almost vertical; two ears	simple	simple	simple
Lyrifissures present	five pairs	five pairs	five pairs	five pairs	three pairs
Disposition notogastral setae	normal	normal	normal	normal	particular

*1* *Congocepheus germani* neotrichy situated at *C* setae alignment

*2* *Congocepheus germani* 8 pairs setiform setae, all situated at neotrichal level

*3* *Cavecarabodes anouchkae* reduction in notogastral setae *C*, with only 12 pairs of setae

The comparison with *Baloghodes* is hard to understand as *Baloghodes* differs greately from *Malgasodes*.

### The posterior prodorsal depression

There are often small differences between immature stases of oribatids during ontogeny, and these differences obey precise rules, for example the number of genital setae is fewer in protonymph than in deuteronymph, which is fewer than in the tritonymph (3 pairs, 4 pairs and 5 pairs), but adult stases differ greatly from immature stases. Studies were not found on ontogenetic development in groups of Carabodidae where the posterior prodorsal depression is present in adult stases, but some exist for *Carabodes* (Grandjean 1949, Andre 1975, Bellido 1978, Ermilov 2011, Reeves 1992) and *Yoshiobodes* (Reeves 1997).

In 1949 Grandjean provided information on orthogenetic development of *Carabodes labyrinthicus*, while immature stases of the same species were studied by Andre in more detail in 1975. Evidently immature stases are very different to adults. Immature stases of Carabodidae are non-sclerotized with soft and fragile bodies. We searched through previous studies to determine if some characteristics could be found in the groups under study.

Species in these studies relate to genera without posterior prodorsal depressions (*p.p.d*), or notogastral anterior depressions (*n.a.d*) in adult stases, but there are very interesting structures on the immature prodorsum of *Carabodes willmanni* Bernini, 1975 and *Yoshiobodes irmayi* (Balogh & Mahunka, 1969) (Reeves 1997).

In *Carabodes willmanni* Bernini, 1975 (Bellido 1978: 423) the prodorsum presents a foveate sclerite situated between lamellar and interlamelar setae (Figure 6, 7 *in* Bellido *op.cit*); this structure can be observed in the larva, but in the protonynph the sclerite is clearly differentiated as a bowl-like structure, and in the deutonymph this zone acquires coloration (considerered melanisation), and sclerotization (Bellido 1978: 423).

Reeves (1997), indicated (page 320) “The scalloped edged depression on the prodorsum of protonymphs, deutonymphs and tritonymphs appears similar to the foveate sclerite found in immature described by Bellido (1978) of *Carabodes willmanni* Bernini.”

In other cases, such as *Carabodes subarticus* (Ermilov 2011 – in russian) the figures of immatures are very deformed, and the presence of the sclerotized field between interlamellar and lamellar setae is only indicated in the description of the deutonymph. On figures 3.1 and 4.1, a structure exists between the interlamellar setae and posterior to the lamellar setae, probably the structure referred to by Bellido and Reeves.

The descriptions of Andre (1975) and Reeves (1992) do not show this structure in immature stases. Bellido (1978: 424) indicated that the existence of this prodorsal microsclerite permits easy differentiation between *Carabodes willmanni* and *Carabodes labyrinthicus*.

We were only able to study adults, not having immatures available, but we consider that the posterior prodorsal depression is probably the same depression found on the immature stases of *Carabodes* and *Yoshiobodes*, and in adult stases on *Congocepheus*, *Bovicarabodes*, *Cavaecarabodes* and *Afticarabodes*.

Another important element is that the prodorsal structure of the prodorsal zone in *Yoshiobodes* was not discussed by the author in text (Figure 9 dorsosejugal region, page 321), similar to *Carabodes interruptus* (Fig. 26) and *Carabodes pentasetosus* (Fig. 34) (in Reeves 1992) where a depression, probably a prodorsal posterior depression of reduced size, exists in the medial region of the prodorsum of the adult, but for these two species no reports on ontogenetic stages exist.

## The anterior notogastral depression

All genera cited in this comparison present an anterior notogastral depression and a particular relation between this depression and notogastral setae.

In genera *Bovicarabodes*, *Afticarabodes*, *Cavaecarabodes* and *Congocepheus* notogastral setae are related to the anterior notogastral depression, but setae are never found inside it; in the case of *Malgasodes*, the situation is very different and four pairs of setae are observed inside the *n.a.d*.

The presence of the notogastral anterior depression and the setal disposition around and/or inside it, involves a very interesting problem overlooked in descriptions of several genera of the family Carabodidae.

In the following genera setae are never found inside the *n.a.d*: *Bovicarabodes*, with *Bovicarabodes deharvengi*, *Bovicarabodes levyi* and *Bovicarabodes fortdauphini*; *Afticarabodes*, with only *Afticarabodes anjavidilavai*; *Cavaecarabodes* with *Ca. pulchritude*, *Ca. hauseri* (Mahunka 1989), *Ca. orientalis* (Mahunka, 1987), *Ca. anouchkae*; *Congocepheus*, with *Congocepheus heterotrichus* Balogh, 1958; *Congocepheus germani*; *Congocepheus gabonensis* Fernandez, Theron, Rollard & Tiedt, 2013; *Congocepheus involutus* Mahunka, 1997; *Congocepheus ektactesi* Fernandez, Theron, Rollard & Tiedt, 2013; and *Congocepheus taurus* Balogh, 1961. Of these *Congocepheus germani* is unique in presenting significant neotrichy (20 pairs of notogastral setae), but setae surround the *n.a.d*, and are never observed inside it.

In *Malgasodes*, the situation is very different. The notogastral anterior depression is present, but the distribution of notogastral setae is unlike all others, with a particular distribution: four pairs of marginal setae *p_1_*, *p_2_*, *p_3_* and *h_3_* in the normal position; four pairs marginal to and two pairs posterior to *n.a.d*; finally four pairs inside *n.a.d*, of which two pairs are close to *d.sj*, these are probably *c_1_* and *c_2_* setae. Lyrifissure numbers are reduced.

The disposition of the notogastral setae is very particular, demonstrating displacement of most of the setae directing to the *n.a.d* (Figure 34), and making accurate notation of the setae near impossible.

**Figure 28–34.**
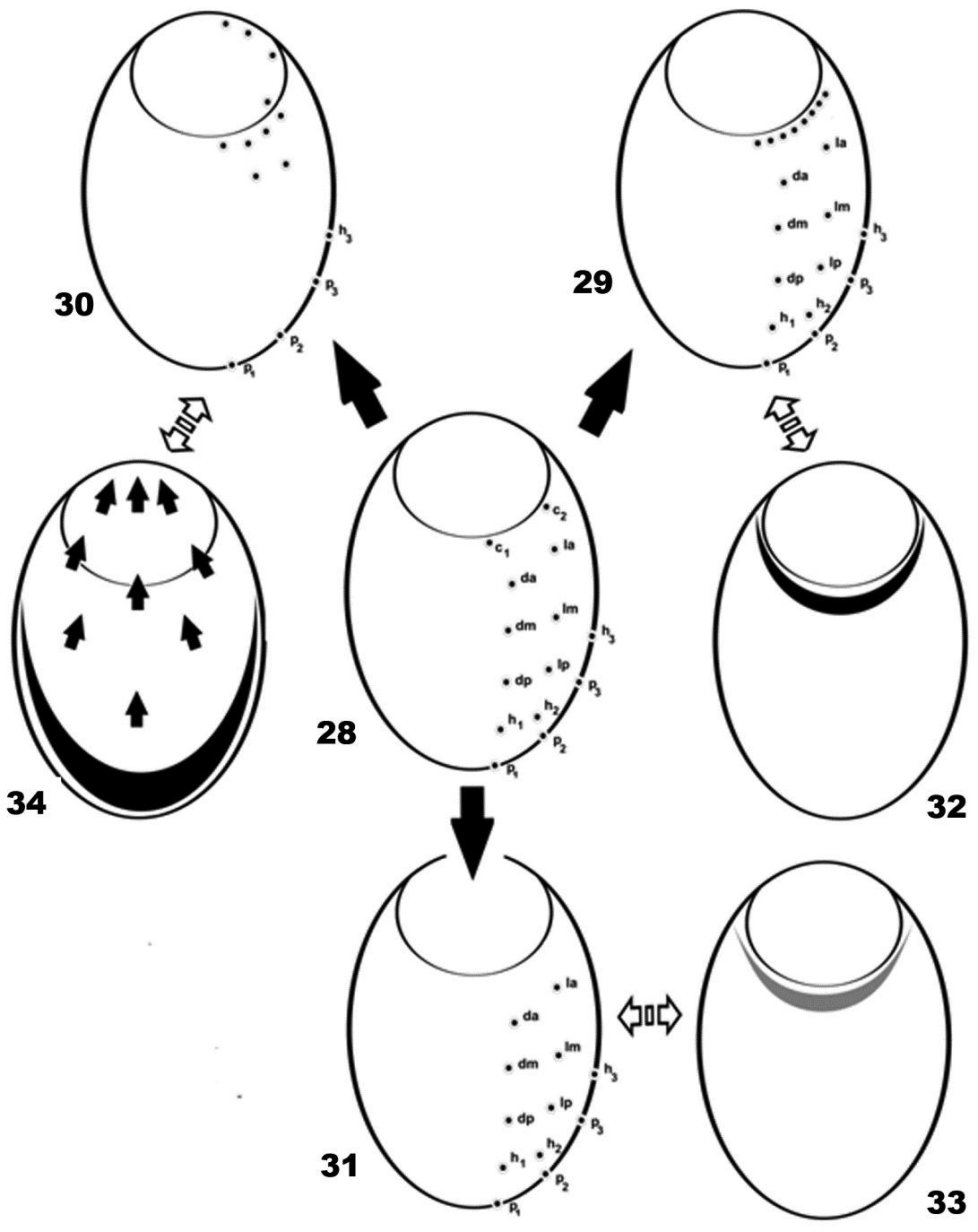
Setal distribution. **28** *Cavaecarabodes*, *Bovicarabodes*, *Afticarabodes*, *Congocepheus* **29** *Congocepheus germani* **30** *Malgasodes curvisetus* **31** *Congocepheus anouchkae* **32** neotrichal zone *Congocepheus germani* **33** regression area of setae *Congocepheus anouchkae* **34** migration process (with arrows) and area of marginal setae *h_3_*, *p_1_*, *p_2_*, *p_3_* of *Malgasodes curvisetus* and *Malgasodes hungarorum*.

We have not been able to advance further, as ontogenetic studies are required; but this displacement process is found near the dorsosejugal furrow (*dis* or *das*) and this may indicate a very interesting evolutionary problem related to segmentation of the opisthosoma, the segmentary origin of the prodorsum and with terminology of the body in general.

### Notogastral setae

The genera *Bovicarabodes*, *Afticarabodes*, *Cavaecarabodes*, *Congocepheus* and *Malgasodes* display holotrichy bideficience (14 pairs of setae) (Figure 8), while in *Malgasodes* the setal distribution is very particular (Figure 30). In some species of *Congocepheus* and *Cavaecarabodes*, the evolutionary process is very interesting; in the case of *Congocepheus* we studied six species: *Congocepheus heterotrichus* Balogh, 1958; *Congocepheus germani* Fernandez, Theron & Rollard, 2014; *Congocepheus gabonensis* Fernandez, Theron, Rollard & Tiedt, 2013; *Congocepheus involutus* Mahunka, 1997; *Congocepheus ektactesi* Fernandez, Theron, Rollard & Tiedt, 2013; *Congocepheus taurus* Balogh, 1961, and several particularities were observed, as discussed in Fernandez et al. (2014). The neotrichy found in *Congocepheus germani* (Figure 29) with a well-established neotrichous territory (Figure 36) (see Fernandez et al. 2014) and the distribution of setae around the border of *n.a.d* is very interesting. In this case setae involved in neotrichy are evidently the alignment *c.*

Another very interesting condition is the reduction process found in *Caveacarabodes anouchkae* (Figure 31) where there are only twelve pairs of setae, with reduction of *c_1_* and *c_2_* setae. The area of setal reduction is indicated in Figure 33.

A completely different situation occurs between the genera *Congocepheus* and *Malgasodes*.

In *Congocepheus* the setae involved in neotrichy are easily identified by a “neotrichous territory” that follows the border of *n.a.d*. However, in the case of *Malgasodes*, a reduction process has occurred where two or three lyrifissures have disappeared. A particular process exists where the setae are directed towards the *n.a.d* (Figure 34), and a large zone of the notogaster is therefore void of setae due to setal migration. Four pairs of setae migrate to inside the *n.a.d*, with two pairs extending to near the *d.sj* furrow; four pairs are found near the border of *n.a.d*; two pairs situated slightly posterior to *n.a.d* and four pairs are situated in the posterior marginal zone of notogaster. Despite no modification in the number of setae (always orthotrichy bideficience) the migration process impedes identification of the setae involved in the process; most probably the two pairs situated near *d.sj* furrow are *c_1_* and *c_2_*, but it is impossible to affirm. The displacement of setae does not involve all of them, because setae *p_1_*, *p_2_*, *p_3_* and h_3_, situated on the marginal zone, are not affected by this process. More complex still, the setae involved are probably from two different segments. The migration process found in *Malgasodes* is very remarkable and necessitates further ontogenetic study.

## Supplementary Material

XML Treatment for Malgasodes

XML Treatment for Malgasodes curvisetus

XML Treatment for Malgasodes hungarorum
